# Fostering better science by releasing biomedical research and innovation from the grip of rich nations

**DOI:** 10.1371/journal.pgph.0004709

**Published:** 2025-06-25

**Authors:** Nathalie J. Arhel

**Affiliations:** CNRS research director, Université de Montpellier, CNRS UMR, Montpellier, France; PLOS: Public Library of Science, UNITED STATES OF AMERICA

In early 2025, the new US administration took unprecedented actions to axe funding to federal science and agencies and suspend foreign aid programs. Beyond the evident toll on global health – such as the closure of clinics that support vulnerable communities and interruptions in epidemiological surveillance – the erosion of US foreign aid presents a potential opportunity to redress long-standing inequities in biomedical research [1,2]. The call for change is not new: the COVID-19 pandemic exposed the vulnerabilities of global health systems, highlighting the importance of national and regional self-sufficiency in research and innovation. Moreover, there is a growing acknowledgement of the need to dismantle systemic power imbalances and confront historical injustices.

By addressing the inequities in biomedical research and considering how these can be redressed, this opinion piece aims to contribute to a broader effort to centre marginalized voices and knowledge systems.

## Inequities in biomedical research

Leadership in scientific research has long been associated with nations of economic and political dominance, shaping who gets to produce knowledge and whose knowledge is valued. In the 19^th^ century, the emergence and dominance of Western science was justified partly by Europe’s military and technological power [3]. Today, most biomedical research and innovation is driven by the world’s largest economies, namely the US and China, who alone contribute to nearly 60% of papers published in high-quality health-science journals, followed by Germany, UK, Japan, France and South Korea whose contributions total ~22% [4]. These countries not only have high gross domestic products (GDP), they also invest proportionately more than other nations in their biomedical research, and create productive systems and infrastructures that nurture discovery and innovation.

Although other countries are equally capable of conducting meaningful biomedical programs that can lead to important discoveries and innovation [5,6], research continues to be severely polarized and driven almost exclusively by institutions from high-income countries (HIC), even when they rely on the resources and infrastructures of lower income countries, such as access to sequencing data or clinical trial capacity [7].

The gap between HIC and the rest of the world (RoW) is fuelled by many factors, including an explicit bias in the scientific literature, dominance of the English language, visa and passport inequities, and prevailing colonial attitudes in how biomedical research is financed and conducted [8–10]. Moreover, since the ability of a researcher to secure grants is dependent on their publication track record, this discrimination creates an ever-widening divide between the top publishing rich institutions, and the RoW.

## Efforts to decolonize biomedical research can reinforce inequities

Ironically, while efforts to be more inclusive of lesser recognised nations might appear beneficial in principle, evidence suggests that they too can contribute to exacerbate patterns of dependency and colonial mindsets.

It is increasingly popular for funding bodies from HIC to devise research grants that are disbursed to their home-grown academics, contingent on partnering with academics from a so-called low-to-middle income country (LMIC). While this has been exemplified as an effective approach to boost international partnerships, these grants can reinforce the exploitation of the know-how and basic materials of resource-rich, yet financially and institutionally poorer, nations for the benefit of global economic powers, and do not generally encourage researchers from these countries to develop their own ideas and take the lead on research projects [11]. They also leave foreign subrecipients at the whim and mercy of their funders who can impose unfair practises with total impunity [12], and withdraw funds without notice as recent events have taught us.

Equally problematic is the issue of foreign-funded research infrastructure and human resources, generally considered an effective mechanism to develop research capacity in lower income nations [7]. When foreign funding bodies establish research or medical facilities without genuine localization, it often leads to a sense of ownership and control by external actors. This can result in a lack of integration with local healthcare systems, limited capacity building, and a perception that these facilities primarily serve the interests of foreign researchers. This dynamic can significantly erode community trust in Western medicine and research, reinforcing historical patterns of exploitation and disregard for local needs and knowledge. Moreover, these investments can be unsustainable on the long run since they assume the country’s government capacity and willingness to sustain operation costs, such as salaries, training, maintenance and supply [13].

Consequently, institutional efforts to promote research in previous colonies, especially when driven by a desire to redress past colonial wrongs or to tap into indigenous resources, can effectively exacerbate the unfair privilege and power of the HIC to drive research on matters in which they have a vested interest. Moreover, sending money to solve the problems of poorer communities, considered by some as “white saviourism”, essentially nurtures a relationship of dependence and perpetuates the unwritten norm that lesser developed countries are incapable of solving their own health problems.

## Towards an inclusive and equitable global governance of biomedical research

**Fig 1:**
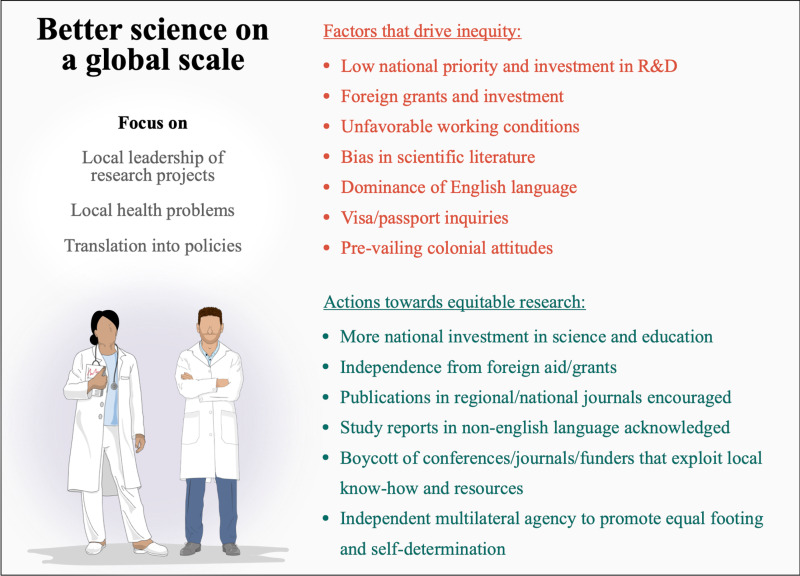
Overview of factors that drive research inequity and actions that would better promote local leadership and equity on a global scale.

In an ideal world, globally equitable biomedical research is one that prioritizes localized research leadership, to generate local knowledge on local health problems, that is translated into policy and practise [14] (Fig 1). Landmark studies that address local health priorities demonstrate that this is possible, as exemplified by the discovery of the haemorrhagic fever vaccine by the Argentinian virologist Julio Barrera Oro [15], which protects against a virus that is only endemic in rural parts of Argentina.

However, many governments lack the resources or political will to prioritize research and development, leaving local scientists struggling for funds and recognition. To begin addressing inequities that arise from disparities in national research and development investment, the international scientific community can take active steps to elevate local leadership, by choosing to publish their research in both national and international journals, appropriately citing regional studies, and integrating local contributions into University curricula and conference programs.

Funding organisations and evaluation committees can also help address inequities by assessing researchers in the context of their working conditions, such as limited national investment in science, unreliable electricity supply, or overbearing teaching responsibilities.

Journals could also drive meaningful change by publishing more studies with exclusively local impact, accompanied by editorial notes to highlight the broader significance of these findings. Entire special issues could be dedicated to locally-led projects, focusing on specific geographical regions.

Above all, individual researchers can help shift the prevailing mindset and hold the global biomedical research infrastructure accountable, through advocacy and by boycotting conferences, journals, and funders that condone exploitative practises and helicopter research. Just as consumers have become a driving force for demanding environmental sustainability from businesses, researchers too can push for greater accountability.

To pilot these changes in a sustainable manner, the governance of global biomedical research could be entrusted to an international multilateral inter-governmental organisation, such as the WHO or Africa CDC. The agency could constitute an autonomous and representative authority of global biomedical research, independently of the currently over-prevalent stakeholders, such as research institutions from HIC, funding bodies and publication houses, who dictate how biomedical research should be done. It could also establish global guidelines on good scientific conduct and provide an opportunity for multilateral discussions of ethical issues, such as genome editing for non-therapeutic purposes or gain-of-function experiments on pathogens.
